# Verticillin A-Loaded Surgical Buttresses Prevent Local Pancreatic Cancer Recurrence in a Murine Model

**DOI:** 10.1021/acs.molpharmaceut.4c00589

**Published:** 2025-01-27

**Authors:** Zeinab Y. Al Subeh, Herma C. Pierre, Cedric J. Pearce, Mark W. Grinstaff, Aaron H. Colby, Kebin Liu, Nicholas H. Oberlies

**Affiliations:** 1 Department of Medicinal Chemistry and Pharmacognosy, Faculty of Pharmacy, Jordan University of Science and Technology, Irbid 22110, Jordan; 2 Department of Biochemistry and Molecular Biology, Medical College of Georgia, Augusta, Georgia 30912, United States; 3 Department of Chemistry and Biochemistry, University of North Carolina at Greensboro, Greensboro, North Carolina 27402, United States; 4 Mycosynthetix, Inc., Hillsborough, North Carolina 27278, United States; 5 Departments of Biomedical Engineering and Chemistry, Boston University, Boston, Massachusetts 02215, United States; 6 Ionic Pharmaceuticals, LLC, Watertown, Massachusetts 02472, United States; 7 Georgia Cancer Center, Medical College of Georgia, Augusta, Georgia 30912, United States; 8 Charlie Norwood VA Medical Center, Augusta, Georgia 30904, United States

**Keywords:** verticillin A, drug-loaded surgical buttress, local drug delivery, prolonged drug release, pancreatic cancer, histone methyltransferase

## Abstract

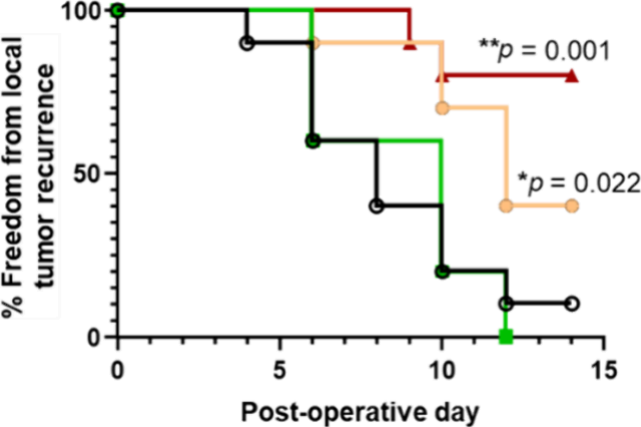

The fungal metabolite verticillin A is a potent and selective histone methyltransferase inhibitor. It regulates apoptosis, the cell cycle, and stress response, and displays potent activity in the suppression of tumor cell growth in several different in vivo models. Verticillin A sensitizes pancreatic cancer cells to anti-PD-1 immunotherapy by regulating PD-L1 expression. However, as with many natural products, delivery and systemic toxicity are challenges that must be overcome to advance their use as a chemotherapeutic. To both reduce systemic toxicity and improve delivery, we report a verticillin A-loaded surgical buttress, which is well-tolerated at a dose as high as 40 mg/kg. In contrast, free verticillin A administered systemically results in toxicity at a dose of 3 mg/kg. The verticillin A-loaded buttress suppresses tumor recurrence in vivo in a safe and dose-dependent manner against a highly aggressive and metastatic model of pancreatic cancer.

## 1 Introduction

Pancreatic cancer is one of the most lethal malignancies and is ranked as the seventh leading cause of cancer-related deaths worldwide.^1,2^ In the United States, it is the third leading cause of cancer death in both genders, with an estimated 5-year survival rate of 13%.^3^ These unfortunate statistics are due, at least in part, to the high recurrence rate among pancreatic cancer patients even after surgical resection (∼84%) or adjuvant chemotherapy postresection surgery (∼87%).^4^ In the case of metastatic pancreatic cancer, median survival is <1 year.^5^

Surgical resection of the primary tumor is the only potentially curative treatment for pancreatic cancer. However, in most cases, surgery alone does not impart long-term survival^6,7^ (e.g., 5-year and 15-year survival rates are ∼16^8,9^ and ∼6%,^10^ respectively). Adjuvant chemotherapy is administered with the goal of preventing tumor recurrence postsurgical resection.^11^ Based on the multicenter randomized controlled ESPAC-4 trial, 6 months of adjuvant chemotherapy with gemcitabine and capecitabine is the current standard treatment for pancreatic cancer patients.^12,13^ In the case of intolerance or toxicity, gemcitabine monotherapy or 5-fluorouracil/folinic acid can be considered.^14,15^ Despite modest improvement in survival rates following adjuvant chemotherapy, high recurrence rates, poor tumor responses, and toxicity are still major concerns for pancreatic cancer patients.^16,17^ Thus, there is a need not only for new drugs, but also, new approaches to treat pancreatic cancer, particularly with an eye toward improving bioavailability and minimizing toxicity.

Targeted drug delivery is one strategy being explored as a means of improving the treatment of pancreatic cancer. Unlike systemic administration of chemotherapeutics, targeted drug delivery aims for local delivery of a chemotherapeutic specifically at the tumor site.^18,19^ This paradigm is intended to both improve efficacy and reduce systemic toxicity.^20,21^ For example, in murine models of pancreatic cancer, tumor-specific delivery of gemcitabine via nanoparticle formulations showed increased efficacy, inhibited tumor growth, and prolonged survival.^22,23^ Similarly, gemcitabine-loaded autologous exosomes exhibited significant suppression of both tumor growth and systemic toxicity in tumor-bearing mice.^24^ In murine xenograft pancreatic cancer models, gemcitabine codelivered with cis-platinum via a biodegradable thermosensitive copolymer hydrogel sustained-release formulation afforded high antitumor efficacy and minimized systemic toxicity.^25^ The targeted delivery of another drug, monomethylauristatin E, via a low molecular weight polymer system, enabled passive accumulation and selective release via extracellular β-glucuronidase, thereby reducing the occurrence of pancreatic tumors and systemic toxicity in murine models.^26^ Tumor resection and recurrence models of targeted pancreatic cancer drug delivery systems have also been explored, albeit not as frequently. Examples include murine models assessing the efficacy of a gemcitabine HCl-loaded microdevice^27^ and a 5-fluorouracil-embedded 3D-printed biodegradable patch,^28^ both of which were successful at suppressing the growth of tumors while minimizing systemic toxicity. In short, while a number of in vivo studies of targeted delivery of drugs to pancreatic tumors have been reported, few have evaluated this in tumor resection models.

A recent review described the discovery, biological activity, and therapeutic potential of the verticillins.^29^ Of note to this report, pharmacological studies over the past decade have repeatedly shown potent cytotoxic activity in vitro against a variety of cancers, including astrocytoma,^30^ breast,^30,31^ cervical,^32^ colon,^33,34^ colorectal,^30^ gastric,^32^ lung,^30^ melanoma,^30,31^ ovarian,^31,35^ and soft tissue sarcoma.^36^ However, investigations into the in vivo anticancer activity of verticillins have been limited to three main studies,^29^ wherein verticillin A was evaluated against ovarian cancer,^35,37^ 5-fluoruracil-resistant colon cancer,^38^ and pancreatic cancer.^39^ The mechanism underlying the anticancer activity of verticillin A is not yet completely understood; however, it is a selective inhibitor of histone methyltransferases that exhibits redundant functions in histone H3 lysine 9 (H3K9) trimethylation and *FAS* transcriptional silencing.^38^ Accordingly, verticillin A treatment decreases H3K9 trimethylation levels and restores *FAS* expression, thereby leading to apoptosis.^38^ In a murine pancreatic cancer model, verticillin A inhibited six histone methyltransferases, allowing for downregulation of histone H3 lysine 4 (H3K4) trimethylation expression, subsequent downregulation of PD-L1 and, ultimately, a higher response to checkpoint immunotherapy.^39^

**Figure 1:**
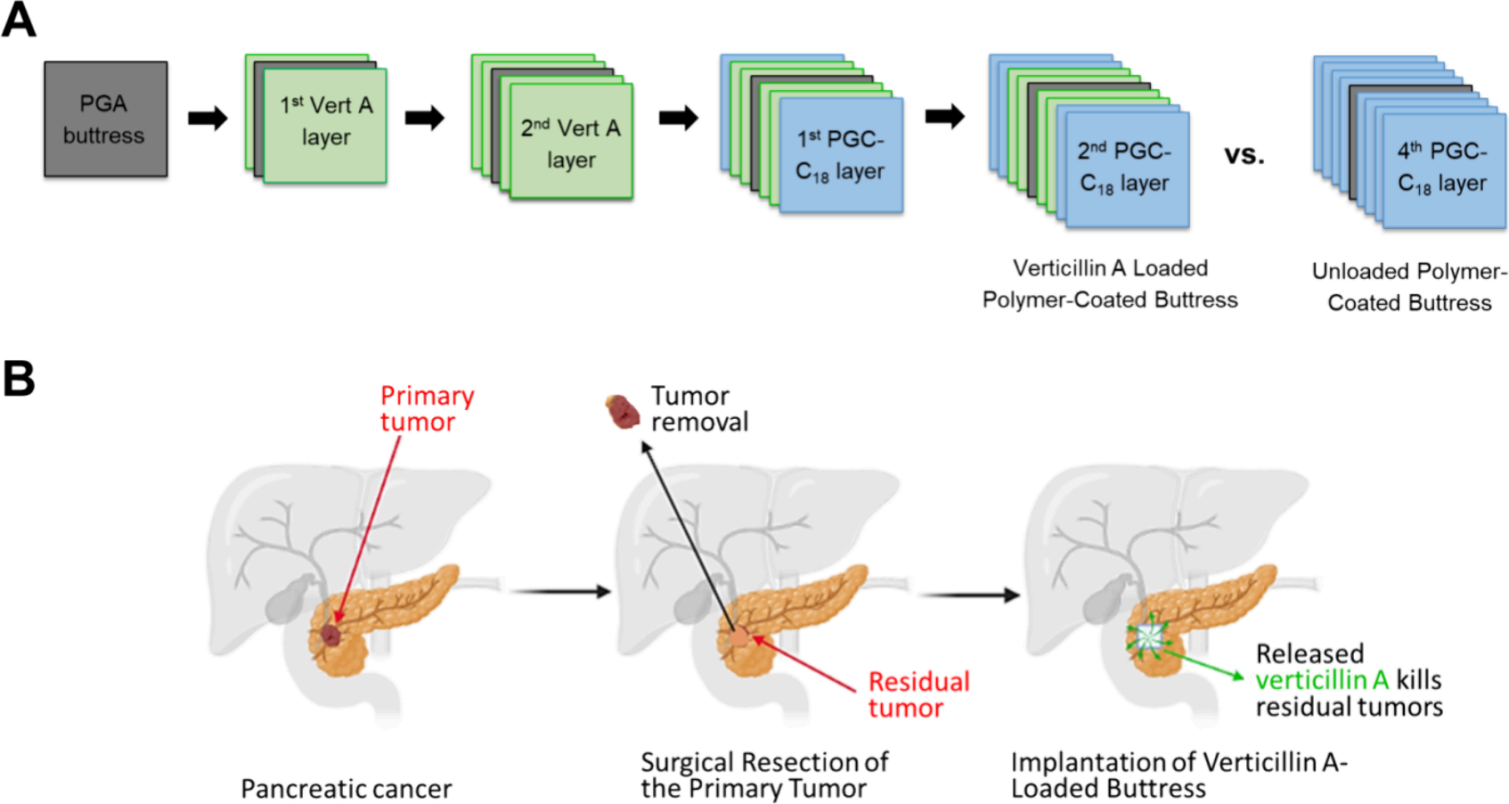
(A) Schematic of the preparation and composition of a verticillin A-loaded surgical buttress. (B) Illustration of pancreatic cancer treatment using a verticillin A-loaded surgical buttress to prevent local recurrence of the disease.

Two significant obstacles with verticillins are liver toxicity and poor water solubility, which makes traditional intravenous delivery challenging.^35,37,40^ As such, studies to address these pitfalls are ongoing, including the development of semisynthetic analogues^31^ and delivery via nanoparticle encapsulation.^35^ Based on promising results with the latter, where loading verticillin A into an expansile nanoparticle system resulted in a modest, though significant, improvement in response while reducing hepatotoxicity in a murine model of ovarian cancer,^35^ we hypothesized that an alternate delivery platform that directly delivers verticillin A locally to the tumor would yield further improvements. This strategy has been used with success previously for paclitaxel^41,42^ and cisplatin,^43^ and to deliver a different fungal metabolite, eupenifeldin, via a drug-loaded surgical buttress to prevent the local recurrence of lung cancer in a murine model.^44^ Adaptation of a delivery system for lung cancer to pancreatic cancer is an attractive strategy not because of phenotypic or histologic similarity between the two cancers but because of the clinical treatment paradigm. In both cases, patients diagnosed with early-stage disease are likely to undergo surgical resection of the tumor, at which point a drug-loaded surgical buttress could be implanted that reinforces the resection margins while also providing the local delivery of a chemotherapeutic. Herein, we report the development and characterization of a verticillin A-loaded, polymer-coated surgical buttress as an extended-release formulation to prevent the local recurrence of pancreatic cancer primary tumor resection (Figure 1).

## 2 Materials and Methods

### 2.1 Materials and Instrumentations

Verticillin A was isolated and characterized from the fungus *Clonostachys rogersoniana* (strain MSX59553) as detailed previously.^30,45^ A Thermo Q Exactive Plus mass spectrometer (ThermoFisher, San Jose, CA, USA) equipped with an electrospray ionization source and connected to a Waters Acquity UPLC system was used to collect HRESIMS-UPLC data in positive mode. The UPLC separations were achieved using a BEH C_18_ column (Waters, 50 mm × 2.1 mm, 1.7 μm) equilibrated at 40 °C and with a flow rate of 0.3 mL/min. The mobile phase consisted of a linear gradient of CH_3_CN:H_2_O (0.1% formic acid) that started at 15:85 and increased to 100:0 over 10 min. ^1^H NMR data were collected by using a JEOL ECA-500 NMR spectrometer (JEOL USA, Inc.) operating at 500 MHz. Chemical shift values were referenced to the residual solvent signal of CDCl_3_ (δ_H_ = 7.26). ^1^H NMR and UPLC analyses were used to assess the purity of verticillin A, which was determined to be >95% (Figures S1 and S2).

Phosphate buffer saline (PBS) was prepared in-house with a pH of 7.35–7.45 and was supplemented with 2% Tween 80 (VWR International) and 0.02% of sodium azide (Sigma-Aldrich) to improve the solubility of verticillin A and prevent microbial growth, respectively. Poly(glycerol monostearate co-ε-caprolactone) polymer (PGC–C_18_) was synthesized as described previously.^46^ Polyglycolic acid polymer (PGA), which is a biodegradable polymer that is used in FDA-approved sutures and buttresses, was utilized to develop a fibrous mesh that served as a buttress onto which PGC–C_18_ polymer was loaded with or without verticillin A. The PGA mesh was formed via electrospinning of a 20% w/v solution of PGA dissolved in hexafluoro-2-propanol, as described previously;^44^ the physical properties are summarized in Table S1 and match commercially available buttress specifications

### 2.2 Preparation and Quantification of Verticillin A

Eight samples with known concentration of verticillin A and dissolved in PBS with 2% Tween 80 were prepared in triplicate in the range of 0.08 to 10.24 μg/mL. The calibration curve of verticillin A was established by injecting these standards in the UPLC-HRESIMS system. For mass spectrometry, the selected ion monitoring (SIM) scanning mode was used to limit the detection to the mass-to-charge ratio (*m*/*z*) range 696.1026–698.1026, which brackets the [M + H]^+^ of verticillin A (i.e., 697.1031 for C_30_H_29_N_6_O_6_S_4_). A BEH Shield RP18 column (Waters, 1.7 μm; 50 × 2.1 mm) heated to 40 °C was connected to the Waters Acquity UPLC system using the aforementioned mobile phase and gradient. The area under the curve was used to build the standard calibration curve (Figure S5), and linear least-squares regression analysis was used to assess its linearity, yielding a linearity range was 0.08–10.24 μg/mL and a correlation coefficient (*R*^2^) of 0.9997. Percentages of the relative error (RE) and the relative standard deviation (RSD) were utilized to evaluate the accuracy and precision, respectively, of the verticillin A calibration curve (Table S2), which had an RE of <15.0%. The limit of quantitation (LOQ), which is the lowest amount of analyte that can be quantitatively determined with suitable precision and accuracy, was 285.1 ng/mL according to the equation (), where *S_a_* is the standard deviation of the *y*-intercept and *b* is the slope of the calibration curve.

### 2.3 Preparation of Verticillin A-Loaded Buttresses

A sheet of PGA polymer was cut into buttresses of two different surface areas; specifically, 1.0 cm^2^ buttresses were used for the in vitro release and long-term cytotoxicity studies, while 0.5 cm^2^ buttresses were used for the in vivo studies. Each buttress was coated with PGC–C_18_ polymer and verticillin A following the layer-by-layer protocol described by Al Subeh et al. (Figure 1A).^44^

Briefly, 1200 μg of verticillin A-loaded buttresses (1 cm^2^) were utilized to study the in vitro release profile of verticillin A in PBS (2% Tween 80). These were prepared by dissolving PGC–C_18_ polymer and verticillin A in HPLC-grade chloroform to produce a clear verticillin A-polymer solution, which was used to coat the PGA buttress with the first layer over the upper and lower faces of each buttress using a Hamilton syringe (Figure 1A). This layer was allowed to dry for at least 1 h before spreading the second layer of verticillin A-polymer solution. After drying overnight, a second solution of PGC–C_18_ in HPLC-grade chloroform was used to add two layers of unloaded polymer on each face of the buttress to cover the verticillin A-polymer layers (Figure 1A). These two layers of PGC–C_18_ polymer were found previously to slow the release rate of the drug-loaded buttress,^44^ and the amounts of PGC–C_18_ and verticillin A loaded into each layer of the verticillin A-loaded buttresses are summarized (Table S3). A similar method was followed to develop high (1200 μg) and low (100 μg) verticillin A-loaded buttresses (1.0 cm^2^) to investigate their long-term cytotoxic properties compared to unloaded buttresses.

A PGA buttress of reduced surface area (0.5 cm^2^) was used in vivo to facilitate subcutaneous implantation on the flank area of the mice (Figure S3). For the maximum tolerable dose (MTD) study, the amount of loaded verticillin A was evaluated over five amounts: 0, 100, 200, 400, and 800 μg. Additionally, high (800 μg) and low (100 μg) verticillin A-loaded buttresses were utilized to study the prevention of the recurrence of pancreatic cancer in vivo compared to unloaded buttresses. The amounts of PGC–C_18_ and verticillin A loaded into each type of verticillin A-loaded buttresses have also been summarized (Table S3).

### 2.4 Release Study of Verticillin A-Polymer Loaded Buttresses

The release profile of verticillin A was monitored over 90 days by submerging a 1200 μg verticillin A-loaded buttress in 50 mL PBS supplemented with Tween 80 (2% v/v) at pH of 7.35–7.45 and 37 °C. To avoid saturation of the buffer solutions and ensure the continuous monitoring of verticillin A release, the PBS media were collected at specific time intervals and replaced with fresh buffer. Collected buffer samples were refrigerated at 4 °C until analysis; a day before analysis, buffer samples were shaken overnight at 37 °C to ensure homogeneity and that verticillin A was fully dissolved. The concentration of verticillin A in collected PBS samples was measured using the UPLC-HRESIMS system, as described above for the calibration curve.

### 2.5 Cytotoxicity of Verticillin A against Pancreatic Cancer Cell Lines

To evaluate the cytotoxicity of verticillin A against murine and human pancreatic cancer cell lines (Panc02-H7 and Miapaca-2, respectively), cells were seeded in 96-well clear flat-bottom plates in a total of 3000 cells per well using RPMI 1640 medium for the former and DMEM medium for the latter. Both media were supplemented with fetal bovine serum (10%), penicillin (100 units/mL), and streptomycin (100 μg/mL). After 24 h at 37 °C and 5% CO_2_, a stock solution of verticillin A dissolved in DMSO (dimethyl sulfoxide) was prepared at 20 mg/mL (i.e., 28.7 mM) and added to the cell culture at various dilutions to have a final concentration range from 0.5 nM to 29.5 μM. For each dilution of verticillin A, a vehicle solution with an equivalent volume of DMSO was used as a control group, and in all cases, the amount of DMSO did not exceed 1% of the total volume of the well. All vehicle controls and verticillin A test groups were performed in triplicate. The cells were then incubated in the presence of verticillin A for 72 h at 37 °C and evaluated for viability using the commercially available tetrazolium-based colorimetric assay of 3-(4,5-dimethylthiazol-2-yl)-5-(3-carboxymethoxyphenyl)-2-(4-sulfophenyl)-2*H*-tetrazolium (MTS; CellTiter 96 Aqueous One Solution Cell Proliferation Assay).^47^

### 2.6 Long-Term Cytotoxicity of Verticillin A-Polymer Loaded Buttresses

The cytotoxicity of 1200 μg and 100 μg verticillin A-loaded buttresses, along with unloaded buttresses (as shown at the right of Figure 1A), were evaluated against murine and human pancreatic cancer cell lines (Panc02-H7 and Miapaca2, respectively) over a period of 10 weeks (Figure S4). These were immersed in 1.5 mL of complete media in a 12-well plate and incubated at 37 °C for 24 h to allow for the release of verticillin A and/or PGC–C_18_ polymer from the buttress into the media. Then, these verticillin A and/or PGC–C_18_ polymer-containing media were transferred into 12-well plates of adherent Panc02-H7 and Miapaca2 cancer cells (5 × 10^4^ cells/well) 24 h after plating. The viability of tumor cells was assessed 48 h after incubation with these samples (Figure S4). Unloaded buttresses (shown at the right of Figure 1A) acted as a vehicle control, while nontreated cells were used as a negative control. Cell viability was assessed using a tetrazolium-based colorimetric assay of 3-(4,5-dimethylthiazol-2-yl)-5-(3-carboxymethoxyphenyl)-2-(4-sulfophenyl)-2*H*-tetrazolium (MTS; CellTiter 96 Aqueous One Solution Cell Proliferation Assay). SoftMax Pro Software was used with a microplate reader. To allow for the continuous release of verticillin A or PGC-C_18_ polymer, the buttresses were immersed in fresh media every 24 h (1.5 mL for each buttress) until the next cycle of cytotoxicity testing. The media for both cell lines are as described in Section 2.5.

### 2.7 In Vivo Maximum Tolerable Dose Study of Verticillin A

A maximum tolerable dose (MTD) study of systemically administered verticillin A was conducted using 8 to 12 week-old C57BL/6 mice. The mice were randomized into five treatment groups (3–4 mice per group), where each animal received one intraperitoneal injection of verticillin A (i.e, 0, 1, 2, 3, and 5 mg/kg), which was solubilized in 10% Cremophor-containing phosphate buffer saline. Animals were monitored over 20 days for any significant weight loss, lethargy, or clinical deterioration.

### 2.8 In Vivo Dose-Escalation Study of Verticillin A-Loaded Buttresses

To determine the optimal dose of verticillin A-loaded buttresses in terms of efficacy vs toxicity, five different doses of verticillin A, i.e, 0 (0 mg/kg), 100 (5 mg/kg), 200 (10 mg/kg), 400 (20 mg/kg), and 800 μg (40 mg/kg), were loaded into 0.5 cm^2^ PGA buttresses. All five formulations of verticillin A-loaded buttresses were coated with equal amounts and layers of PGC–C_18_ polymer (Table S3). The reduced size of the PGA buttresses (i.e., 0.5 cm^2^ instead of 1 cm^2^; Figure S3) was to allow for more convenient implantation on the flank areas of the animals.

Subcutaneous implantations of the five doses of verticillin A-loaded buttresses were performed on the right flank of 8 week-old nontumor-bearing C57BL/6 mice (5 animals per group). Animal weight and survival were followed over 30 days. Humane euthanasia was implemented if there was a significant drop in body weight (i.e., > 20%).

### 2.9 In Vivo Murine Model of Local Pancreatic Cancer Recurrence

To determine the efficacy of verticillin A-loaded buttresses in preventing the recurrence of pancreatic cancer, 8 week-old female C57Bl/6 mice were injected subcutaneously in the right flank with murine pancreatic cancer cells (Panc02-H7; 500,000 cells/animal). After 2 weeks, the primary visible tumors were then resected under isoflurane anesthesia, leaving behind adjacent tissue with residual cancer cells to allow for tumor regrowth. Animals were randomized into four treatment groups: (1) implantation of unloaded PGC–C_18_-coated buttress, (2) implantation of 100 μg of verticillin A-loaded buttress, (3) implantation of 800 μg of verticillin A-loaded buttress, and (4) intraperitoneal injection of gemcitabine (50 mg/kg/week). The buttresses were placed directly over the resection bed, but underneath the skin flap, and the incisions were closed with wound clips. With this design, drug release from the buttress is intended to be bidirectional. The animals were monitored for local recurrence of the tumor and weight loss over 14 days. Animals were euthanized at the end of the study, and the secondary tumor was resected and weighted. Statistical analyses were carried out using GraphPad Prism software. A log-rank (Mantel-Cox) test was used to assess the efficacy of the treatments for statistical significance in prolonging tumor-free periods.

### 2.10 Histological Analysis

Livers and kidneys of the euthanized animals were harvested and stored in 10% formalin, followed by paraffin embedding, sectioning, and H&E staining.

## 3 Results

### 3.1 Kinetics of Verticillin A Release

**Figure 2:**
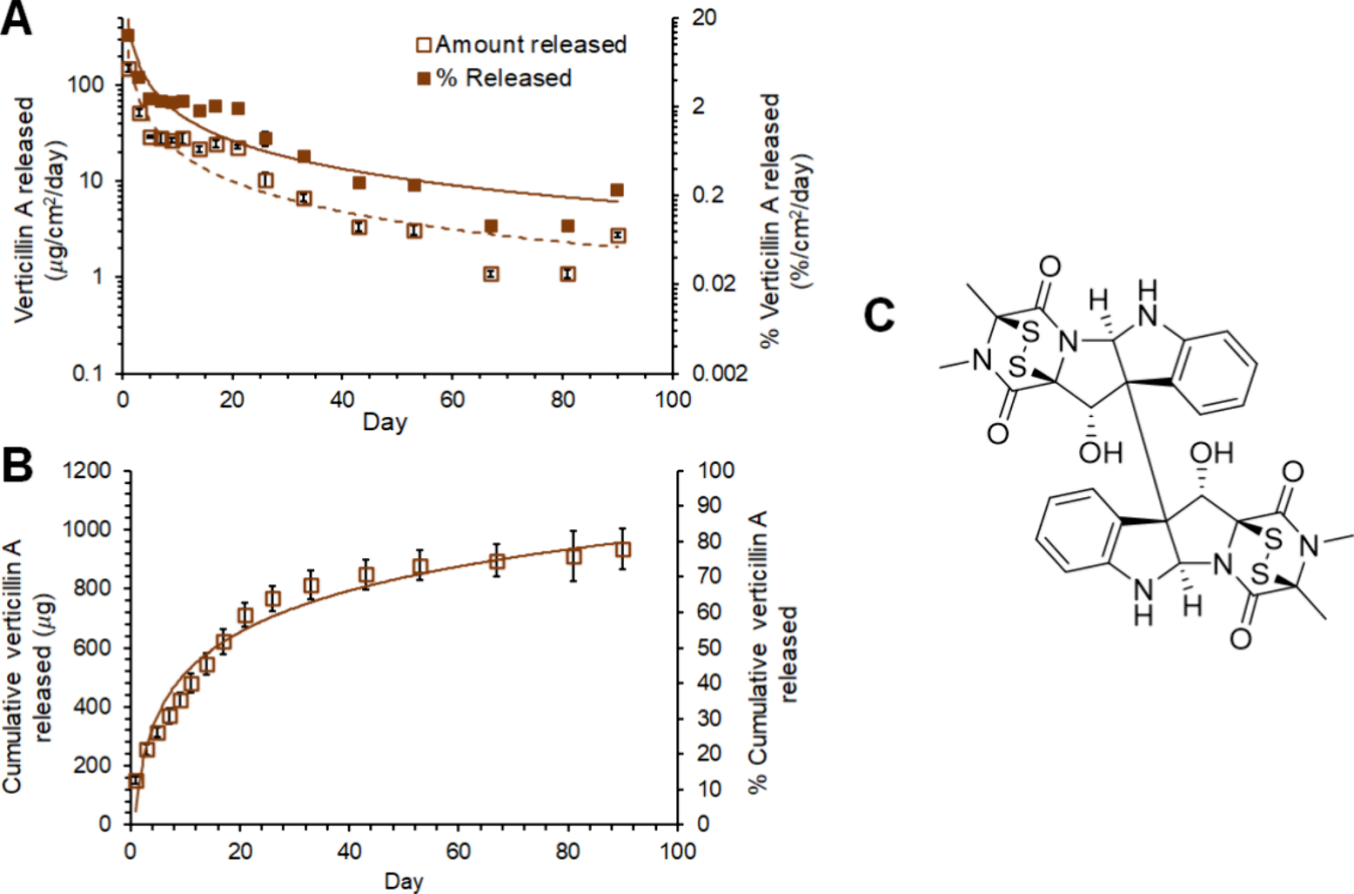
Kinetics of verticillin A release. (A) Daily release rate of verticillin A normalized by the surface area of the loaded buttress and plotted as the amount (i.e., μg) released/cm^2^/day (Dashed line) and percent released/cm^2^/day (solid line). (B) Cumulative release profile of verticillin A-loaded buttress over 90 days plotted as the mass of verticillin A (μg, left side) and percent (%, right side) of total loaded verticillin A in the buttress (i.e., 1200 μg). (C) Structure of verticillin A (C_30_H_28_N_6_O_6_S_4_). For plots (A) and (B), each time point represents three experimental replicates. Error bars represent the standard deviation.

The prevention of local tumor recurrence can be achieved by employing a system that delivers therapeutic, nontoxic doses of treatments over extended timeframes.^44^ Previously, we used a strategy in which a PGA surgical buttress, which is used clinically, was coated with a solution of the fungal natural product eupenifeldin and PGC-C_18,_ a biocompatible, biodegradable, hydrophobic polymer, to achieve loading and metered, extended drug release.^44^ Using the formulation with the most linear and sustained release profile achieved in that study (i.e., PGA buttress coated with 1200 μg of drug-loaded PGC-C_18_ followed by two coats of unloaded PGC-C_18_ polymer), we characterized the release profile of verticillin A over 90 days (Figure 2).

The release profile of verticillin A-loaded surgical buttresses (Figure 2B) demonstrated a limited burst release with only 12% in the first 24 h. The formulation continued to release a moderate amount of verticillin A for approximately 21 days (Figure 2A) before reaching a plateau, at which point 60% was released. The formulation then maintained a relatively consistent extended release of verticillin A up to the 90-day mark, at which point ∼80% of the total verticillin A initially loaded was released.

### 3.2 Cytotoxicity of Verticillin A-Polymer Loaded Buttresses against Pancreatic Cells In Vitro

**Figure 3:**
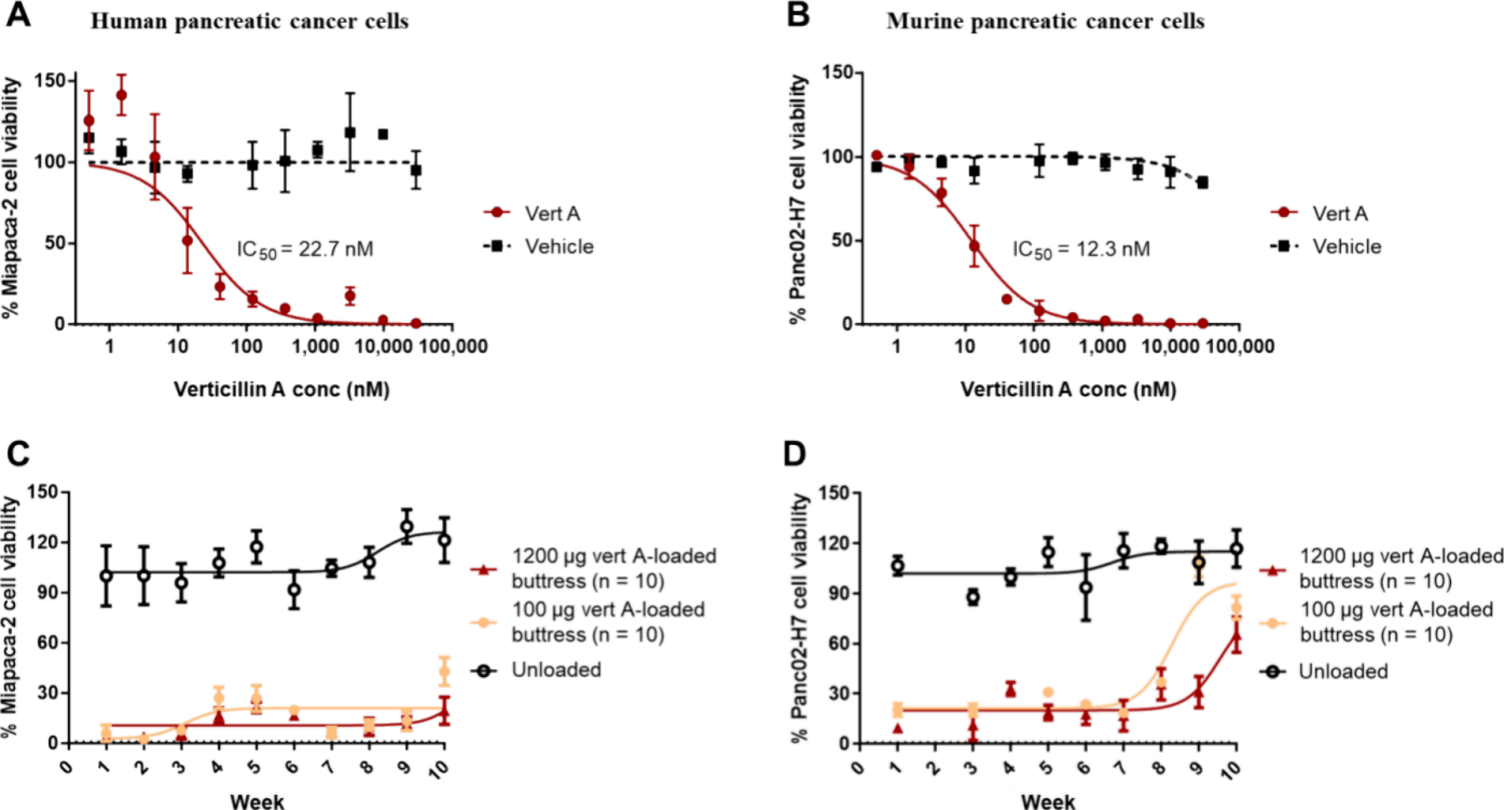
In vitro cytotoxicity of verticillin A, either alone (A, B) or after release from a verticillin A-loaded surgical buttresses (C, D), against human (A, C) and murine (B, D) pancreatic cancer cells (Miapaca2 and Panc02-H7, respectively). (A) IC_50_ value of verticillin A against human pancreatic cancer cells (Miapaca-2). (B) IC_50_ value of verticillin A against murine pancreatic cancer cells (Panc02-H7). (C) Long-term cytotoxicity of verticillin A after release from 1200 and 100 μg verticillin A-loaded buttresses against human pancreatic cancer cells (Miapaca2) over 10 weeks. (D) Long-term cytotoxicity of verticillin A after release from 1200 and 100 μg verticillin A-loaded buttresses against murine pancreatic cancer cells (Panc02-H7) over 10 weeks. Note, in both panels (C) and (D), the unloaded buttress represents the buttress shown in the diagram at the right of Figure 1A, where no test compound was loaded. Table S3 provides more details about how verticillin A was loaded onto each buttress.

Verticillin A, tested as a single agent, was cytotoxic against both human (Miapaca-2) and murine (Panc02-H7) pancreatic cancer cell lines with IC_50_ values of 22.7 and 12.3 nM, respectively (Figure 3A,B). We then assessed the long-term cytotoxicity of the verticillin A-loaded buttresses in both pancreatic cancer cell lines (1200 and 100 μg of verticillin A per buttress; *n* = 10 per sample and cell line). Cytotoxicity was maintained for 10 weeks in human cell lines and 7–9 weeks in murine pancreatic cancer cell lines incubated with release media from 1200 and 100 μg verticillin A-loaded buttresses (Figure 3C,D). As a comparator, the data for an unloaded buttress are shown as well.

### 3.3 Dose-Escalation and MTD Definition of Systemically Administered Verticillin A vs Verticillin A-Loaded Surgical Buttresses

**Figure 4:**
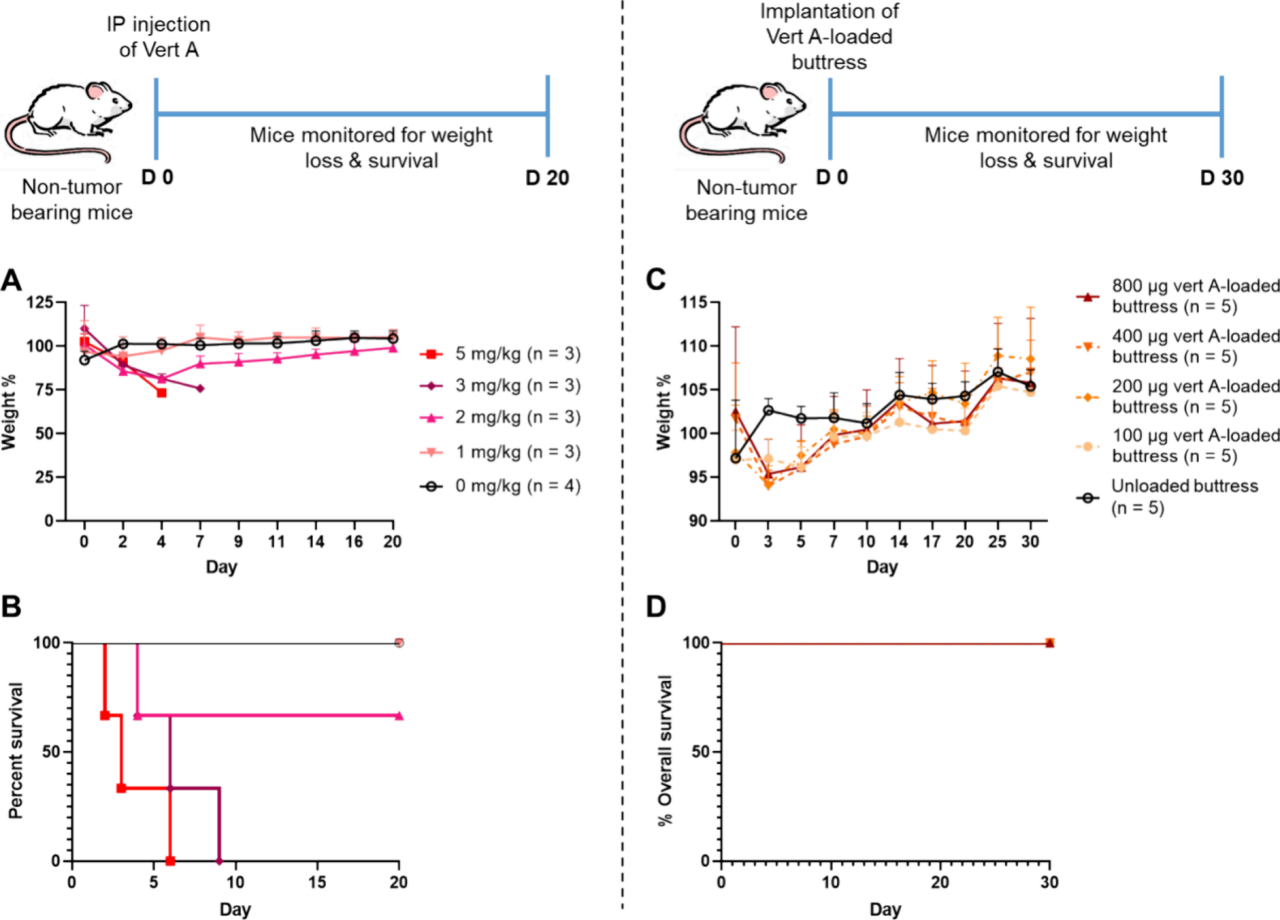
Maximum tolerable dose (MTD) study of systemically administered verticillin A vs dose-escalation study of verticillin A-loaded surgical buttresses using nontumor bearing mice. (A, B) Percent of animal’s weight change and survival plotted over 20 days after intraperitoneal injection of verticillin A in a dose range of 0–5 mg/kg. (C, D) Percent of animal’s weight change and survival over 30 days after subcutaneous implantation of verticillin A-loaded surgical buttresses with a dose range of 0–800 μg (i.e., 0 to 40 mg/kg). In all panels, *n* represents the number of mice per treatment group.

To determine the MTD of systemically administered verticillin A, nontumor-bearing mice (*n* = 16) received single IP injections of “free” verticillin A (i.e., solubilized in Cremophor EL/ethanol); this was designed to mimic the clinical formulation of paclitaxel (i.e., Taxol).^48,49^ Animals received doses of 0 (*n* = 4), 1 (*n* = 3), 2 (*n* = 3), 3 (*n* = 3), and 5 mg/kg (*n* = 3). All animals receiving verticillin A doses of 3 or 5 mg/kg experienced severe (≥25%) weight loss (Figure 4A) and, ultimately, early mortality (Figure 4B). In the 2 mg/kg group, only one animal experienced severe weight loss (Figure 4A) and early mortality (Figure 4B); however, after day 4, the surviving mice (2 out of 3) improved and sustained their weights. Therefore, the MTD of free verticillin A was determined to be 2 mg/kg.

To determine the MTD of verticillin A-loaded surgical buttresses, five different doses were prepared and administered to a total of 25 nontumor bearing mice (*n* = 5 buttresses per dose). The amount of verticillin A loaded onto the 0.5 cm^2^ PGA buttresses were 0 (unloaded), 100 (5 mg/kg), 200 (10 mg/kg), 400 (20 mg/kg), and 800 μg (40 mg/kg) (Table S3). The mice in all treatment groups maintained stable body weights (Figure 4C) and survived for a minimum of 30 days (Figure 4D). These data established that the MTD of verticillin A-polymer loaded buttresses was >800 μg (i.e., 40 mg/kg).

### 3.4 In Vivo Efficacy of Verticillin A-Polymer Loaded Buttresses

**Figure 5:**
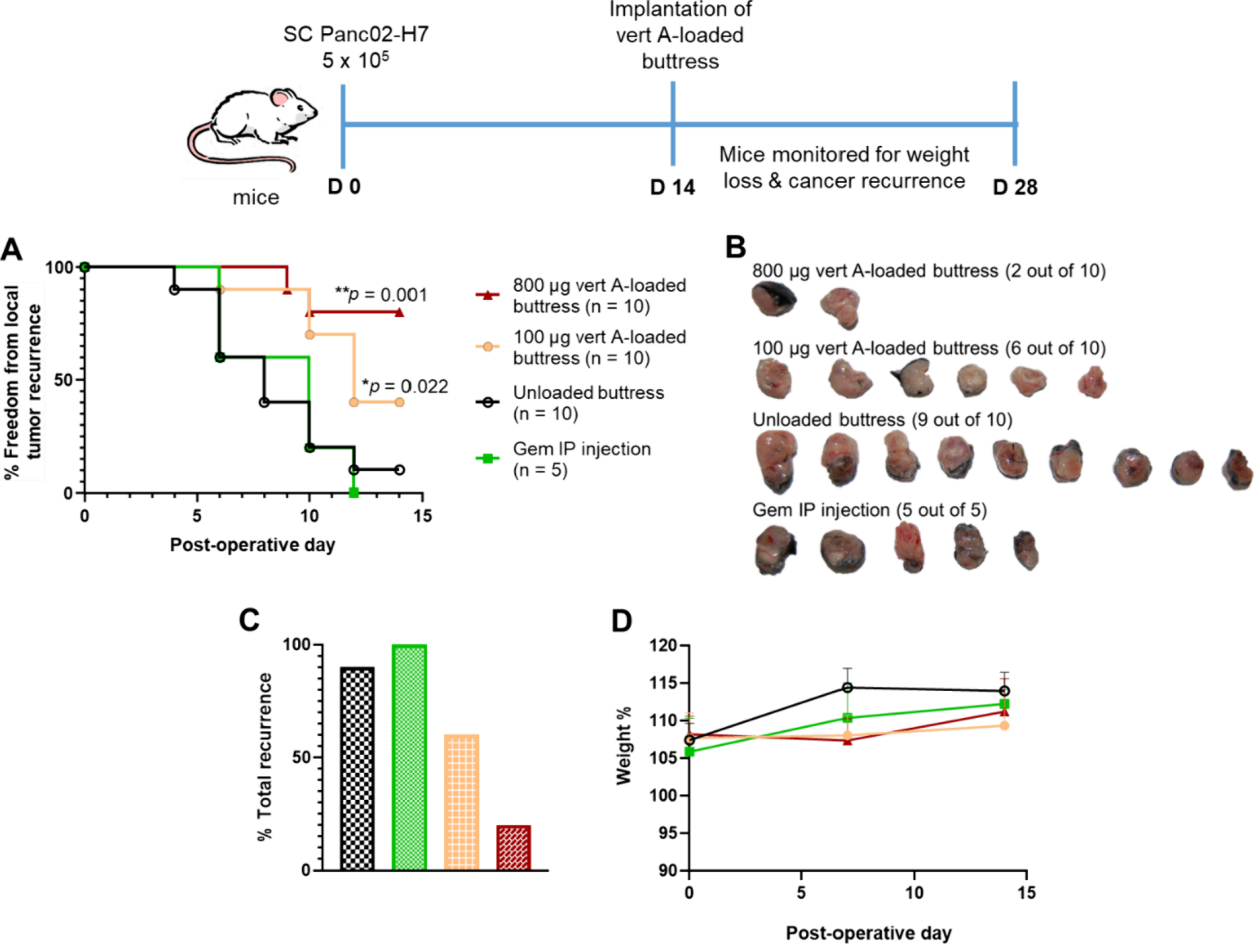
In vivo efficacy study of verticillin A-loaded surgical buttresses. (A) Percent of freedom from local tumor recurrence over 14 days after subcutaneous implantation of 100 μg and 800 μg verticillin A-loaded buttresses (i.e., equivalent to 5 and 40 mg/kg, respectively). (B) Images of the recurred tumors among the treatment groups after 14 days. (C) Percent of tumor recurrence among the treatment groups. (D) Percent of animals’ weight change among the treatment groups over 14 days. In panel (A), *n* represents the number of mice per treatment group. Note, the color coding in panels (C) and (D) follows the key shown in panel (A).

To assess the in vivo efficacy of verticillin A-loaded surgical buttresses in the prevention of pancreatic cancer tumor recurrence, a subcutaneous murine model was employed. Tumors were established by injecting Panc02-H7 cells in the right flank of C57BL/6 mice (*n* = 35). Two weeks post xenografting, primary tumors were resected, the mice were randomized, and the following treatments were administered: gemcitabine IP injection (50 mg/kg/week, positive control, *n* = 5), verticillin A-loaded surgical buttress at both 800 μg (40 mg/kg, *n* = 10) and 100 μg (5 mg/kg, *n* = 10), or 0 μg (i.e., unloaded or negative control, *n* = 10). For the duration of the experiment, the mice in all treatment groups maintained stable body weights, but the tumor recurrence rates differed. Animals treated with a gemcitabine IP injection had a 100% tumor recurrence rate by day 12, whereas mice treated with 100 μg (i.e., equivalent to 5 mg/kg) verticillin A-polymer loaded buttresses showed a significant (*p* < 0.05), 40%, reduction in tumor recurrence at this same time point. Animals receiving 800 μg (i.e., equivalent to 40 mg/kg) of verticillin A-loaded surgical buttresses demonstrated an even greater (80%, *p* = 0.001) reduction in tumor recurrence compared to IP gemcitabine at the same time point (Figure 5A).

### 3.5 Histological Analysis

**Figure 6:**
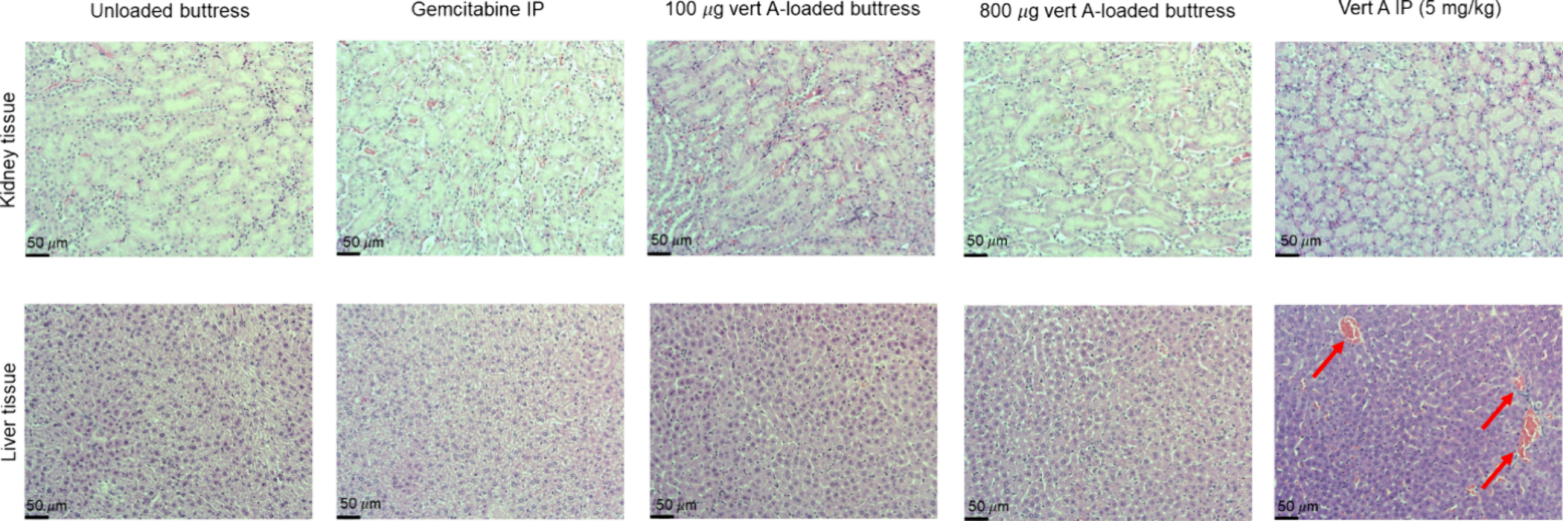
Histological analysis of harvested liver and kidney tissue of mice treated with gemcitabine or verticillin A (polymer and IP administration). All tissue samples were comparable to the control (unloaded polymer-coated buttresses), except for the liver tissue of mice treated with verticillin via IP (bottom right). The lesions in the tissues (indicated with red arrows) are indicative of hepatotoxicity.

Following the study end point, animal livers and kidneys were harvested, stored in 10% formalin, and processed for histopathological analysis. Mice treated with free verticillin A had liver lesions, while those treated with gemcitabine or verticillin A-polymer-loaded buttresses had normal histopathological results (Figure 6).

## 4 Discussion

Pancreatic cancer is one of the most intractable cancers worldwide.^39,50,51^ Given the high recurrence rate among pancreatic cancer patients post-tumor resection,^11^ new approaches are needed. As such, we investigated the use of the fungal metabolite verticillin A^29^ as a potential pancreatic cancer chemotherapeutic. However, like many natural products, verticillin A exhibits poor solubility and hepatotoxicity.^31,35,37^ To mitigate these drawbacks, we developed a verticillin A-loaded drug delivery system to provide prolonged and controlled release over an extended 90 day period to eliminate residual pancreatic tumors and prevent tumor recurrence. Herein, we assessed the efficacy of this drug delivery system including the kinetics of release, long-term cytotoxicity, maximum tolerable dose, and in vivo efficacy.

The drug release profile of the verticillin A-loaded surgical buttresses mirrored that of our most effective drug-polymer-loaded buttress formulation from our eupenifeldin system.^44^ There was an expected, though modest, initial burst release of verticillin A that ultimately plateaued from days 21 to 90. These data demonstrate that the verticillin A-loaded buttress is an effective means of delivering consistent therapeutic concentrations of verticillin A to the local target tissue for over 90 days.

The in vitro efficacy of verticillin A-loaded surgical buttresses was evaluated via a long-term cytotoxicity study in human (Miapaca-2) and murine (Panc02-H7) metastatic pancreatic cancer cell lines. The two extremes of the verticillin A-loaded surgical buttresses (i.e., 1200 μg and 100 μg), which were chosen based on previous experience studying a different fungal metabolite in a similar drug delivery study,^44^ maintained long-term, potent cytotoxicity for 8–10 weeks, indicating their suitability as a depot for verticillin A. Prolonged release over such an extended period is desired due to the slow doubling rate of some cancer cells. By providing therapeutic concentrations of the drug over several months, this system is more likely to kill these slow-growing cells, thereby preventing tumor recurrence.

Interestingly, the maximum tolerated dose (MTD) of verticillin A-loaded surgical buttresses is greater than 800 μg (40 mg/kg), which is more than 25 times greater than the MTD of “free” verticillin A solubilized in Cremophor EL/ethanol. This significant increase in safety profile demonstrates both the utility of this particular drug delivery system as well as the potential, more generally, to modify the kinetics, bioavailability, and toxicity of therapeutic agents by altering the route of delivery. Given the known hepatotoxicity observed with verticillin A,^35^ the mortalities were likely caused by liver damage. To confirm this, both livers and kidneys were harvested and underwent histopathological assessments, revealing lesions in the livers of mice treated with “free” verticillin A but not the verticillin A-loaded surgical buttresses. Thus, the drug-loaded surgical buttresses successfully mitigate the hepatotoxic effects of verticillin A and demonstrate that the extended-release mechanism effectively administers verticillin A at therapeutic doses. This is particularly noteworthy given that the amount of verticillin A on the buttress is 25 times higher than what was administered IP.

Given this, both high, 800 μg (i.e., 40 mg/kg), and low, 100 μg (i.e., 5 mg/kg), doses of the verticillin A-loaded surgical buttress were evaluated in an in vivo cytoreductive surgery model using a subcutaneous murine tumor cell line (Panc02-H7), which has been shown to be a highly aggressive and metastatic model of pancreatic cancer.^52^ Both high and low doses result in significant reductions in tumor recurrence rates (80% and 40% reduction compared to the control, respectively). The overall recurrence rates (20 and 60% for high and low doses, respectively) are significantly lower than those for unloaded surgical buttresses (90%) and gemcitabine IP injections (100%).

## 5 Conclusions

Verticillin A-loaded polymer-coated buttresses afford extended release of verticillin A and prevent tumor recurrence in vivo with no observable toxicity. These findings support the use of drug-loaded polymeric buttresses as an adjuvant to standard-of-care surgical resection, and we demonstrate for the first time the potential of this technology for the targeted administration of chemotherapeutics to the resection sites of pancreatic tumors. Studies to further probe the limits of this delivery platform are warranted, particularly with an eye toward tumors where cytoreductive surgery is considered a key part of the treatment strategy and for molecules that have potent cytotoxicity but may suffer from poor bioavailability.

Due to the symmetric “sandwich” design of the buttress (Figure 1A), drug releases bidirectionally (i.e., out of both sides of the buttress). In vivo, interstitial and lymphatic fluid (and even blood) accumulate in the resection “pocket” into which the buttress is placed. Therefore, the entirety of the buttress is wetted and is expected to release bidirectionally. A gross indication of unintended side effects on the side facing away from the resection bed (i.e., the skin side) would be the observation of impaired wound healing or necrosis in the skin flap above the buttress (i.e., due to a high concentration of drug in a thin, poorly vascularized tissue). However, this was not observed in any animals and suggests that verticillin A release and accumulation in the skin flap were not toxic. Further studies are ongoing to advance the testing of verticillin A in vivo against a variety of tumor models.

## Supporting Information Available

^1^H NMR spectrum of verticillin A; UPLC chromatogram of verticillin A; physical properties of PGA buttresses; precision and accuracy of verticillin A calibration curve; amounts of verticillin A and PGC-C_18_ polymer used for the in vitro and in vivo studies; image of verticillin A-loaded surgical buttress next to a US penny; protocol for evaluating long-term in vitro cytotoxicity of verticillin A-loaded surgical buttress; calibration curve for measuring verticillin A by UPLC-HRESIMS (PDF)
