# Supplementary material for: Verticillin A-Loaded Surgical Buttresses Prevent Local Pancreatic Cancer Recurrence in a Murine Model
Source: Mol Pharm. 2025 Jan 27;22(3):1220–9. doi: 10.1021/acs.molpharmaceut.4c00589 (PMC11881038; doi:10.1021/acs.molpharmaceut.4c00589)
Supplement: Supplementary file 1 — mp4c00589_si_001.pdf [file mp4c00589_si_001.pdf]

Supporting Information

Verticillin A- Loaded Surgical Buttresses Prevent Local Pancreatic Cancer Recurrence in a Murine Model

Zeinab Y. Al Subeh,[1,2,#] Herma C. Pierre,[3,#] Cedric J. Pearce,[4] Mark W. Grinstaff,[5] Aaron H. Colby,[5,6] Kebin Liu,[2,7,8] Nicholas H. Oberlies,[3,*]

[1]Department of Medicinal Chemistry and Pharmacognosy, Faculty of Pharmacy, Jordan University of Science and Technology, Irbid 22110, Jordan.

[2]Department of Biochemistry and Molecular Biology, Medical College of Georgia, Augusta, Georgia 30912, United States.

[3]Department of Chemistry and Biochemistry, University of North Carolina at Greensboro, Greensboro, North Carolina 27402, United States.

[4]Mycosynthetix, Inc., Hillsborough, North Carolina 27278, United States.

[5]Departments of Biomedical Engineering and Chemistry, Boston University, Boston, MA 02215, United States.

[6]Ionic Pharmaceuticals, LLC, Watertown, MA 02472, United States.

[7]Georgia Cancer Center, Medical College of Georgia, Augusta, Georgia 30912, United States.

[8]Charlie Norwood VA Medical Center, Augusta, Georgia 30904, United States.

*Corresponding author: Nicholas H. Oberlies (nicholas_oberlies@uncg.edu)

Supporting Information Content

Figure S1. ^{1}H NMR Spectrum and Labeled Structure of Verticillin A in $CDCl_3$ [500 MHz]. 3
Figure S2. **A.** UPLC chromatogram of verticillin A, demonstrating > 95% purity. **B**. (+)-HRESIMS spectrum of verticillin A. 4
Figure S3. Verticillin A- loaded surgical buttress (0.5 cm^2) next to a US penny for size comparison. 8
Figure S4. The protocol followed to evaluate the long-term in vitro cytotoxicity of the 1200 µg & 100 µg verticillin A-loaded surgical buttresses. 9
Figure S5. Standard calibration curve of verticillin A established using UPLC-HRESIMS system. 10

Table S1.Physical properties of the developed PGA buttresses. 5
Table S2. Precision (RSD%) and accuracy (RE%) of verticillin A calibration curve. 6
Table S3. Amounts of verticillin A and PGC-C_{18} polymer loaded on the PGA buttresses that were utilized for the in vitro and the in vivo studies. 7

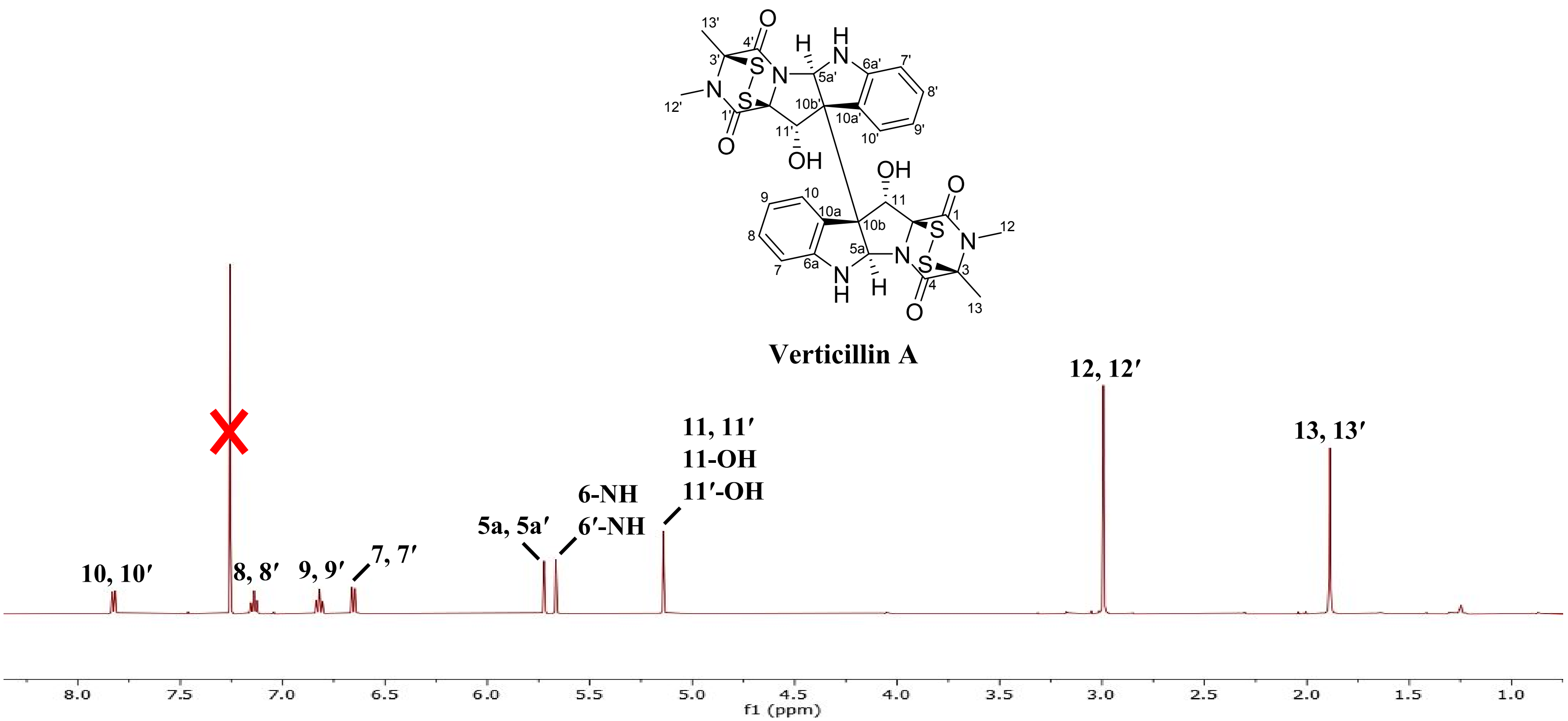

Figure S1. ^{1}H NMR Spectrum and Labeled Structure of Verticillin A in $CDCl_3$ [500 MHz].

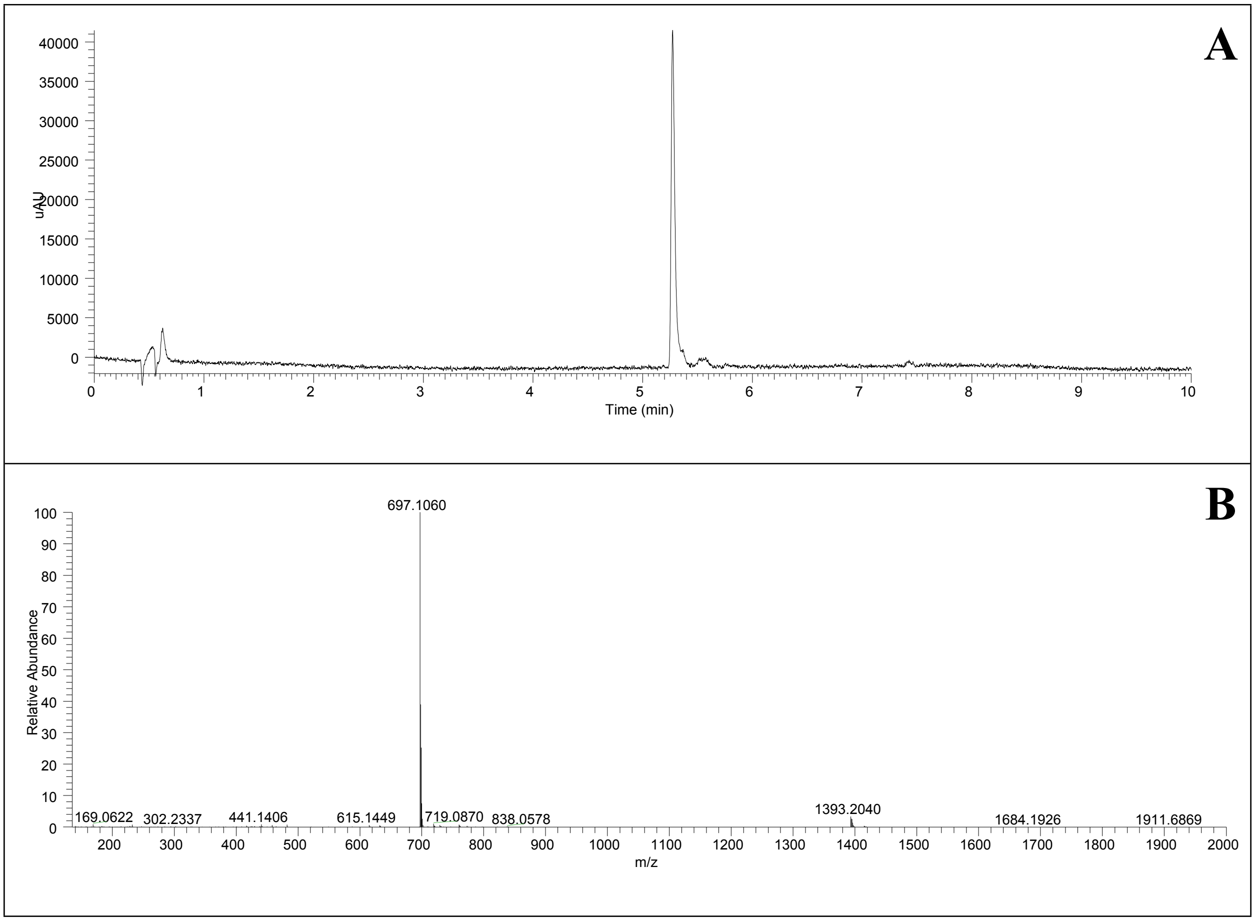

Figure S2. **A.** UPLC chromatogram of verticillin A, demonstrating > 95% purity. Data were acquired via Acquity UPLC system using an Acquity BEH Shield RP18 column (Waters, 1.7 µm; 50 × 2.1 mm) equilibrated at 40°C with a flow rate set to 0.3 mL/min. The gradient system consisted of CH_3CN/H_2O in 0.1% formic acid and increased linearly from 15 to 100 % CH_3CN over 7 min. **B**. (+)-HRESIMS spectrum of verticillin A. Data were acquired using a ThermoFisher Q Exactive mass spectrometer (ThermoFisher, San Jose, CA, USA) with a heated electrospray ionization source.

Table S1. Physical properties of the developed PGA buttresses.

Thickness	~ 140 μm
Mass density	5.84 g/cm^2
Mean fiber diameter	3.63 μm
Porosity	38.05%

Table S2. Precision (RSD%) and accuracy (RE%) of verticillin A calibration curve.

Concentration of standard solutions (ng/mL)	RSD (%)	RE (%)
80	7.7	-5.5
160	0.1	-11.8
320	1.9	-10.1
640	0.9	-6.2
1280	0.1	-0.6
2560	0.5	2.0
5120	2.3	2.3
10240	0.7	-0.7

RSD%: Relative standard deviation percentage.

RE%: Relative error percentage.

Table S3. Amounts of verticillin A and PGC-C_{18} polymer loaded on the PGA buttresses that were utilized for the in vitro and in vivo studies.

Verticillin A-loaded buttresses utilized in the in vitro kinetic study					
Type	Amount of loaded vert A (μg)	Amount of vert A per layer (μg)	Amount of loaded PCG-C_{18} (mg)	Amount of loaded PCG-C_{18} per layer (mg)	Buttress size (cm^2)
1200 μg vert A-loaded buttresses (Equivalent to 60 mg/kg)	1200	300	24	3	1
Verticillin A-loaded buttresses utilized in the in vitro cytotoxicity studies					
Type	Amount of loaded vert A (μg)	Amount of vert A per layer (μg)	Amount of loaded PCG-C_{18} (mg)	Amount of loaded PCG-C_{18} per layer (mg)	Buttress size (cm^2)
100 μg vert A-loaded buttresses (Equivalent to 5 mg/kg)	100	25	24	3	1
1200 μg vert A-loaded buttresses (Equivalent to 60 mg/kg)	1200	300	24	3	1
Blank polymer-loaded buttresses (Equivalent to 0 mg/kg)	0	0	24	3	1
Verticillin A-loaded buttresses utilized in the in vivo studies					
Type	Amount of loaded vert A (μg)	Amount of vert A per layer (μg)	Amount of loaded PCG-C_{18} (mg)	Amount of loaded PCG-C_{18} per layer (mg)	Buttress size (cm^2)
100 μg vert A-loaded buttresses (Equivalent to 5 mg/kg)	100	25	12	1.5	0.5
200 μg vert A-loaded buttresses (Equivalent to 10 mg/kg)	200	50	12	1.5	0.5

400 μg vert A-loaded buttresses (Equivalent to 20 mg/kg)	400	100	12	1.5	0.5
800 μg vert A-loaded buttresses (Equivalent to 40 mg/kg)	800	200	12	1.5	0.5
Blank polymer-loaded buttresses (Equivalent to 0 mg/kg)	0	0	12	1.5	0.5

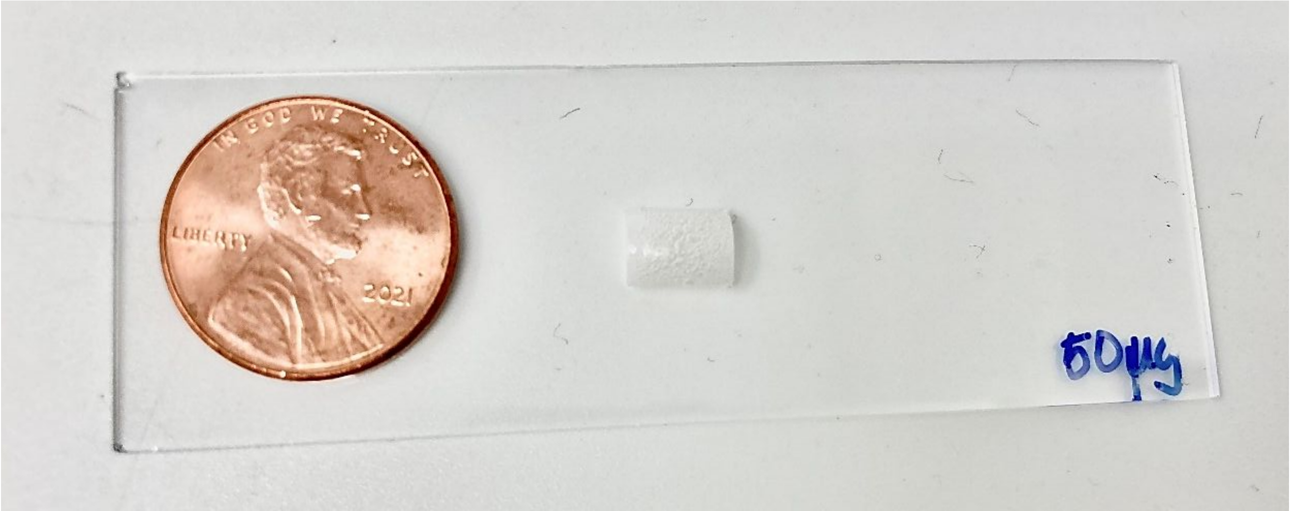

Figure S3. Verticillin A- loaded surgical buttress (0.5 cm^2) next to a US penny for size comparison.

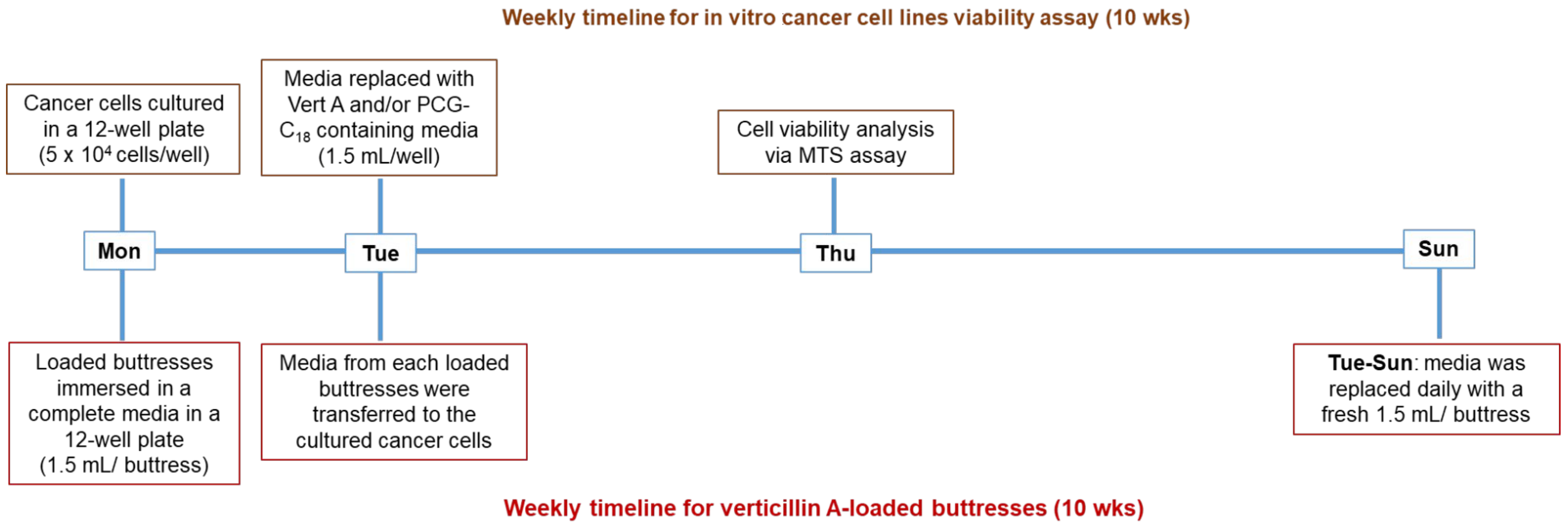

Figure S4. The protocol followed to evaluate the long-term in vitro cytotoxicity of the 1200 μg & 100 μg verticillin A-loaded surgical buttresses.

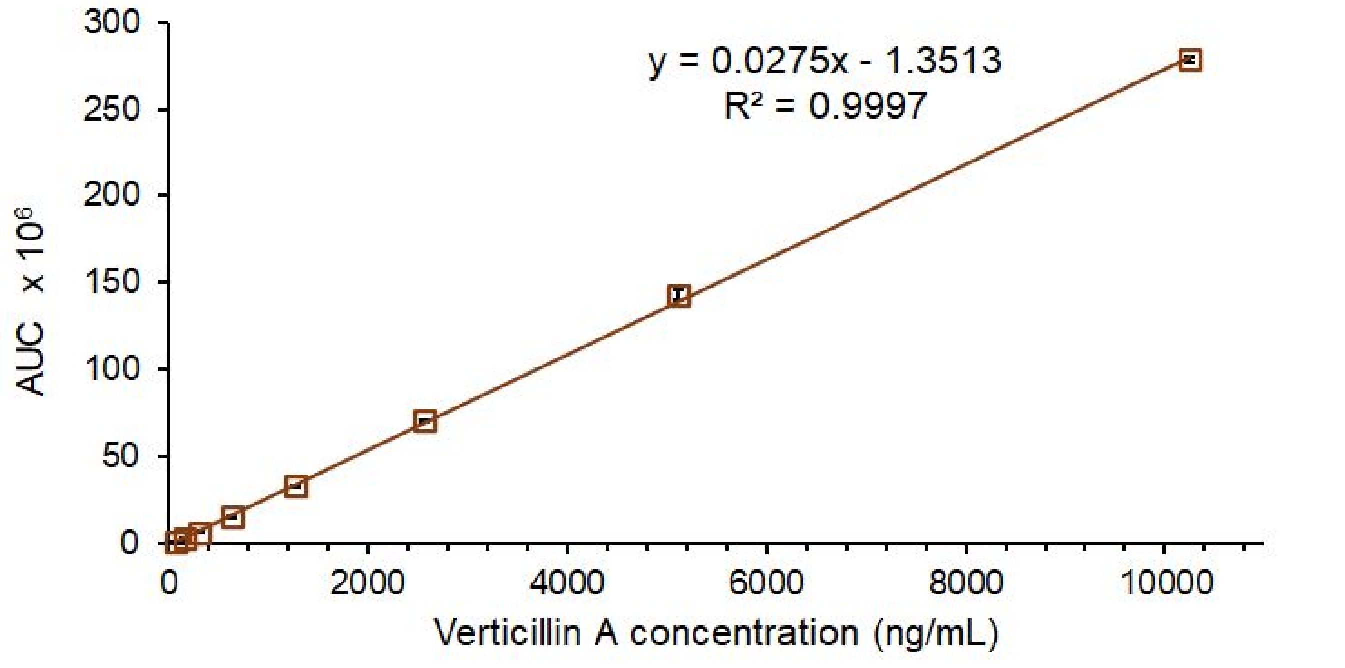

Figure S5. Standard calibration curve of verticillin A established using a UPLC-HRESIMS system.
